# Cesarean Section Due to Social Factors Affects Children's Psychology and Behavior: A Retrospective Cohort Study

**DOI:** 10.3389/fped.2020.586957

**Published:** 2021-01-25

**Authors:** Xiu-Yu Shi, Jing Wang, Wei-Na Zhang, Meng Zhao, Jun Ju, Xiao-Yan Li, Qian Lu, Bin Wang, Li-Ping Zou

**Affiliations:** ^1^Department of Pediatrics, The First Medical Center, Chinese PLA General Hospital, Beijing, China; ^2^Department of Pediatrics, Beijing Friendship Hospital, Capital Medical University, Beijing, China; ^3^Department of Pediatrics, Beijing Chao-Yang Hospital, Capital Medical University, Beijing, China; ^4^Department of Neurology, Capital Institute of Pediatrics, Beijing, China; ^5^Center for Brain Disorders Research, Capital Medical University, Beijing Institute for Brain Disorders, Beijing, China

**Keywords:** cesarean section, elective cesarean section, children, behavior, psychology

## Abstract

**Background:** Cesarean section (CS) use has reached a frequency well-above what is expected on the basis of obstetric indications. The large increase in CS use, often for non-medical indications, is of concern given the risks for both women and children. Research about the influence of CS on children's behavior is not new, but most studies didn't differentiate CS due to social factors (such as fear of labor pain, auspicious dates, etc.) from CS with medical indications. Medical indications for CS include fetal distress and intrauterine hypoxia, which may also affect the mental and physical health of the children, thus be a confounding factor. In China, a significant proportion of women undergo CS because of social factors, which provides us a good model to study whether non-fetal triggered delivery will affect children's behavior. Thus, we assessed the impact of CS due to social factors on child psychology and behavior.

**Methods:** We conducted a retrospective cohort study. Children were divided into three groups according to delivery mode: vaginal delivery (VD), CS with medical indications, and CS due to social factors (also called as elective cesarean section, ECS). Parents or guardians were required to complete four rating scales of Chinese version [Conners' Parent Rating Scale (CPRS), Child Behavior Checklist-Parent Form (CBCL-PF), Swanson, Nolan, and Pelham rating scale-Parent Form (SNAP-IV-PF), and Behavior Rating Inventory of Executive Function-Parent Form (BRIEF-PF)] on psychological and behavioral problems regarding their children.

**Results:** Among the 38,780 children aged 7–15 years, 29,103 (75.05%) were delivered by VD and 9,677 (24.95%) were delivered by CS (7,844 with medical indications; 1,833 by ECS). Ten covariates were found to significantly affect ECS. Four rating scales were used in this study: CPRS, CBCL-PF, SNAP-IV-PF, and BRIEF-PF. ECS affected child psychology and behavior in several aspects including inattention, hyperactivity/impulsivity, social problems, and executive dysfunction. Regarding to inattention, the ECS group had a higher SNAP-IV-PF inattention score (*P* = 0.03), compared with the VD group. Logistic multivariate stepwise regression analysis showed that in the ECS group, the ORs were 1.20 in the partially adjusted analyses of SNAP-IV-PF and CPRS. Regarding to social problems, ECS group had a higher CBCL-PF score for the social problems category compared with the VD group (*P* = 0.0001). Kruskal–Wallis rank sum tests showed that the ECS group had higher BRIEF-PF scores regarding Working Memory (*P* = 0.04), and Organize (*P* = 0.01) compared with the VD group.

**Conclusions:** CS affected the offspring's psychology and behavior. After removing possible influence of medical indications, the effect of CS due to social factors on the offspring's psychology and behavior still exists.

## Introduction

Vaginal delivery (VD) is a natural process which is a culmination of full maturation of the fetus, however, delivery mode has been deeply influenced by social factors in the last few decades. Comparing to Western populations, Asian women are more likely to choose CS. This disparity could partly be attributed to racial characteristics and cultural beliefs. Data from the World Health Organization (WHO) reveal that 46% of births in China involved CS in 2007–2008 (1). High frequency of CS was also found in other countries. In 2018, Boerma et al. (2) reported that frequency of CS in the Latin America and Caribbean region was 44.3%, in the Dominican Republic was 58%. This is the highest rate worldwide and three times higher than the WHO's recommendation of 15%. Historically, the primary reasons to perform CS included obstetric complications or serious maternal illness, in order to save the mother or fetus. More recently, a significant proportion of women undergo CS because of social factors, such as fear of labor pain, concerned about complications (urinary incontinence and lower quality of sex life after VD), misconception of CS being safer and faster than VD, and auspicious dates (3).

The high prevalence of CS—especially CS due to social factors has initiated a debate about whether CS is always appropriate and has drawn attention to the effects of CS on maternal health. Many studies (4, 5) have suggested that CS is associated with an increased risk of complications as well as substantial economic burden. CS also affects the offspring's health, with previous studies showing that CS is associated with asthma, allergic rhinitis, diabetes (6, 7) and childhood acute lymphoblastic leukemia (8), and it may also affect neurological development which may lead to neurodevelopmental disorders (9, 10). Neurodevelopmental disorders usually affect behavioral, learning, and cognitive abilities, which may reduce the quality of life of individuals and their families. For example, attention deficit hyperactivity disorder (ADHD) is the most common neurodevelopmental disorder in children and is characterized by attention difficulty, hyperactivity, and impulsivity. ADHD affects an estimated 5.9–7.1% of children and adolescents worldwide (11) and often persists into adulthood (12). ADHD both directly impairs health and is associated with anxiety, other mood disorders, antisocial behavior, and addiction (13). Furthermore, ADHD leads to lost productivity, high social costs regarding health care and education, and increased conviction rates (14). Talge et al. (15) found that children delivered by CS exhibited higher inattention and ADHD index scores compared with children delivered by VD, especially among males. A link between CS and increased odds of autism spectrum disorder (ASD) has also been suggested (16).

However, interpretation of previous results is limited by small sample and inclusion criteria. For example, most previous studies assessing the effects of CS included CS with medical indications and CS due to social factors. Medical indications for CS include fetal distress and intrauterine hypoxia, which may also affect the mental and physical health of the children. CS due to medical factors is sometimes difficult to prevent, however, prevalence of ECS can be potentially modified by public education. Thus, it's crucial to study the effect of ECS on child behavioral outcomes. In China, a significant proportion of women undergo CS because of social factors, which provides us a good model to study whether non-fetal triggered delivery will affect children's health. Focusing on this group appears to be more appropriate because this differentiates the effects of infant and maternal diseases from CS. Two recent studies reported conflicting results with regards to outcomes of the impact of ECS on childhood behavioral development. Kelmanson reported that children delivered by ECS are more likely to exhibit behavioral and emotional disturbances (17), whereas Li et al. found that children delivered by ECS are less likely to have emotional or behavioral problems (18). Considering the high prevalence of CS in China and conflicting outcomes, it is essential to further explore the long-term effects of CS, particularly CS due to social factors, on child psychology and behavior.

## Methods

### Study Participants

**Figure 1:**
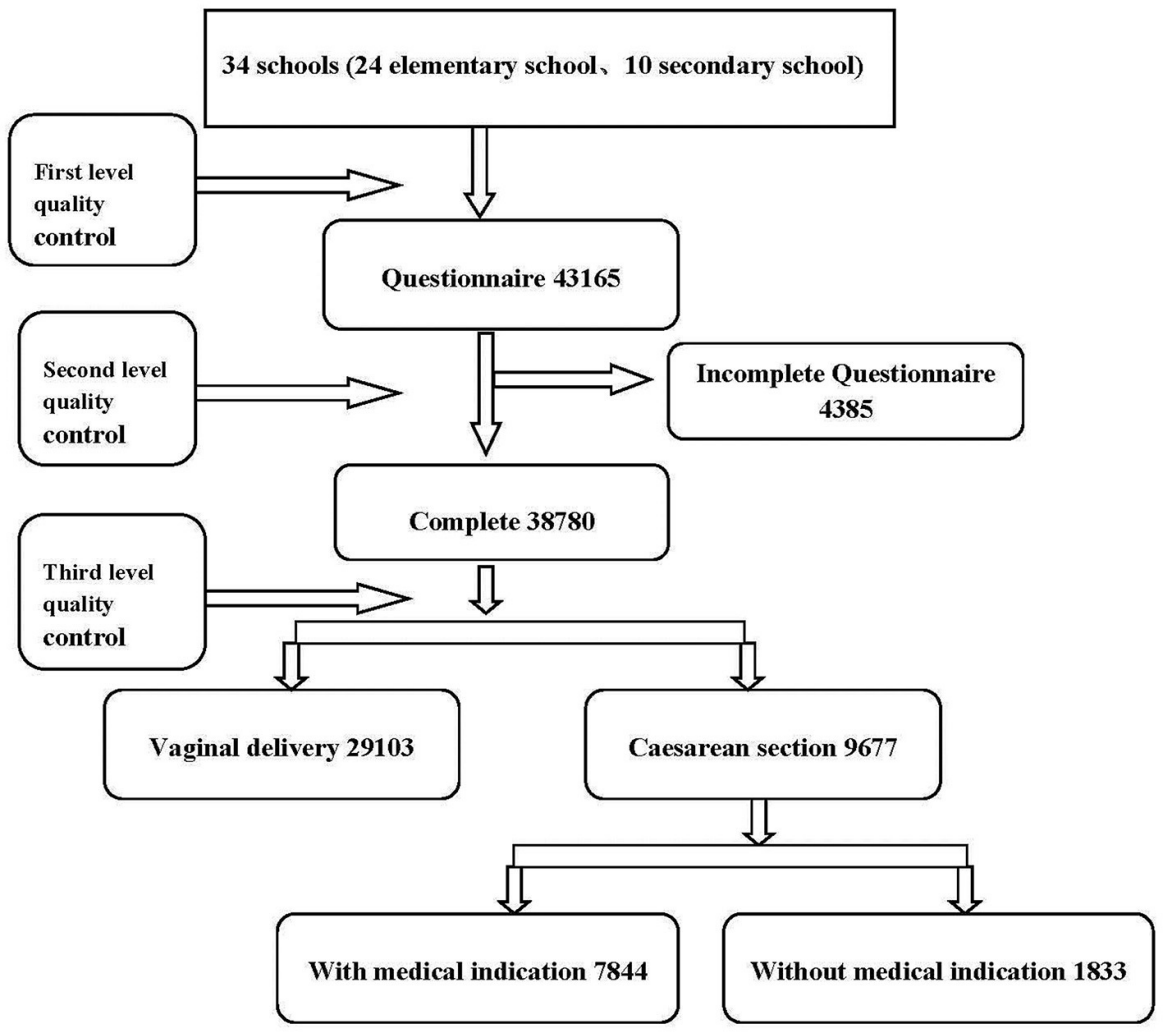
Participant flow chart.

With the help of the Hohhot City Board of Education and the Ethics Committee, students from the second grade of primary school to the third grade of junior high school (7–15 years old) (24 primary schools and 10 junior high schools) whose households were registered in Hohhot were recruited for this study. A total of 43,165 questionnaires were distributed, and 38,780 completed questionnaires (Figure 1).

### Exposure Variable: Delivery Mode

Delivery mode was divided into three categories: VD, CS with medical indications, and ECS. VD included spontaneous VD, induced VD, and assisted VD with the use of forceps or vacuum extraction. Medical indications of CS included fetal distress, cephalopelvic disproportion, breech presentation, transverse lie position, maternal complications, previous CS, and other unspecified indications. ECS was defined as CS due to social factors (fear of labor pain, fear of hurt to baby, misconception of CS being safer and faster than VD, auspicious dates, and other).

### Covariates

Covariates included parental occupations, parental ages, parental education levels, family type (two-parent or single-parent family), quality of family relationships, living conditions, habitable area, total household income; child's age, child's sex (male/female), physical examination, dietary habits, age at kindergarten enrollment; whether the mother had fever during pregnancy, whether abortion was considered, nutritional supplement use, complications, smoking, gestational age, birth weight, Apgar score, asphyxia, umbilical cord around the neck, and jaundice.

### Assessment of Child Psychopathology

#### Conners' Parent Rating Scale (CPRS)

CPRS (19) is a 48-item questionnaire based on a four-point (0, 1, 2, 3) scale according to symptom frequency. There are several subscale scores: conduct problems, impulsivity/hyperactivity, learning problems, anxiety, psychosomatic problems (including headache, stomach pain, other aches, vomiting or nausea, and bowel problems), and total scores.

#### Child Behavior Checklist-Parent Form (CBCL-PF)

CBCL-PF (20) contains 120 items on emotional, behavioral, and social problems reported by parents of children ages 4–16 years. Items related to eight syndrome categories (comprising anxious/depressed, withdrawn/depressed, somatic complaints, social problems, thought problems, attention problems, rule-breaking behavior, and aggressive behavior) are scored as 0 (not true), 1 (somewhat or sometimes true), or 2 (very true or often true) (21). Along with the total CBCL-PF score, scores were calculated for internalizing problems (withdrawn, somatic complaints, and anxious/depressed) and externalizing problems (rule-breaking behavior and aggressive behavior).

#### Swanson, Nolan, and Pelham Questionnaire, 4th Version–Parent Form (SNAP-IV-PF)

SNAP-IV-PF (22) includes 26 items based on a four-point (0, 1, 2, 3) scale according to symptom frequency. It consists of two ADHD symptom subscales, one for inattention (nine items) and one for hyperactivity/impulsivity (nine items), and one subscale for oppositional defiant disorder (eight items). If the score on any of those subscales is 13 or above, it meets the threshold for ADHD diagnosis.

#### Behavior Rating Inventory of Executive Function-Parent Form (BRIEF-PF)

BRIEF-PF (23) is an 86-item questionnaire that is completed by the caregiver according to symptom frequency (0, 1, 2). BRIEF-PF is designed to assess executive functioning in children and adolescents. It consists of the following subscales: Inhibit, Shift, Emotional Control, Working Memory, Plan/Organize, Organization of Materials, Monitor, and Initiate. These subscales yield three composite measures: Behavioral Regulation Index, Metacognition Index (MI), and Global Executive Composite. Higher BRIEF-PF scores indicate more severe executive function impairments.

### Statistical Analyses

After examination of the completeness of the questionnaires, the questionnaire responses were coded, using EpiData software, we developed a CHECK program, and the data were independently inputted twice. To test the reliability and validity of the different rating scales, we used Split-half reliability and Pearson linear correlation analyses. Non-parametric (Kruskal–Wallis) rank sum tests were used for univariate analysis, and Logistic multivariate stepwise regression analysis was performed to control for confounding factors. All analyses were conducted using SAS 9.1 software (SAS Institute, Cary, NC, USA). Statistical significance was set at *P* < 0.05.

## Results

### Distribution of Maternal and Child Characteristics Among the CS, ECS, and VD Groups

**Figure 2:**
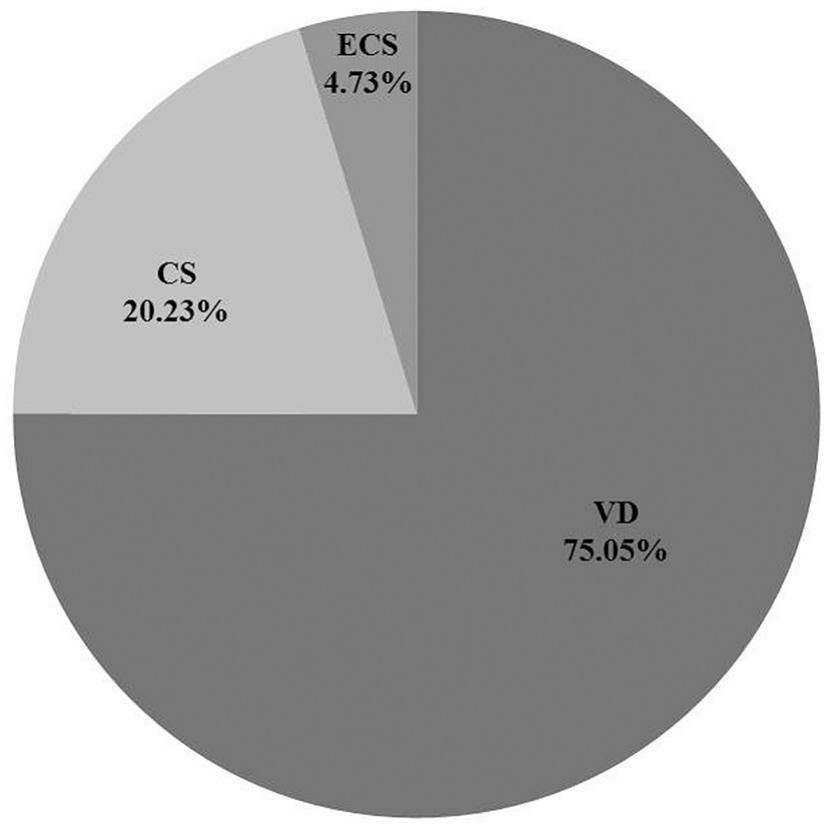
Percentage of different delivery mode. CS, cesarean section with medical indications; ECS, cesarean section due to social factors; VD, vaginal delivery.

A total of 43,165 questionnaires were distributed, 38,780 of which were valid (representing 89.84% of the questionnaires). Among the 38,780 children aged 7–15 years (birth years ranging from 1995 to 2003), 29,103 (75.05%) were delivered by VD and 9,677 (24.95%) were delivered by CS (7,844 with medical indications and 1,833 involving ECS, Figure 2). The distribution of maternal education, child's sex, child's age, living conditions, habitable area, and household income differed significantly by group (*P* < 0.05, Table 1). Ten covariates were found to significantly affect ECS, including higher education, certain occupations (teacher, doctor, and public servant), single-parent family, maternal age, living in building, and etc. (Table 2).

**Table 1:** Maternal and children characteristics according to mode of delivery.

**Characteristics**	**Mode of delivery**			***P* (χ^2^)**
	**VD *n* = 29,103 (%)**	**CS*n* = 7,844 (%)**	**ECS *n* = 1,833 (%)**	
**Mother**				
Education				
University or higher	4,251 (14.6)	1,846 (23.5)	466 (25.4)	<0.0001 (1,142.4)
Junior college	12,863 (44.2)	4,136 (52.7)	989 (54.0)	
Junior high school or less	11,989 (41.2)	1,862 (23.7)	378 (20.6)	
Single birth				
Yes	19,804 (68.1)	5,930 (75.6)	1,349 (73.6)	<0.0001 (180.1)
No	9,298 (31.9)	1,914 (24.4)	484 (26.4)	
**Child**				
Gender				
Male	14,019 (48.2)	4,143 (52.8)	928 (50.6)	<0.0001 (54.9)
Female	15,084 (51.8)	3,701 (47.2)	905 (49.4)	
Age (years)				
7 11	16,703 (57.4)	5,093 (64.9)	1,302 (71.0)	<0.0001 (250.8)
12 15	12,400 (42.6)	2,751 (35.1)	531 (29.0)	
**Family**				
Family type				
Traditional family	28,438 (97.7)	7,674 (97.8)	1,773 (96.7)	0.02 (8.3)
Single parent	665 (2.3)	170 (2.2)	60 (3.3)	
Quality of family relationships				
Harmonious	26,535 (91.2)	7,233 (92.2)	1,680 (91.7)	0.01 (8.6)
Average or poor	2,568 (8.8)	611 (7.8)	153 (8.3)	
Living conditions				
Building	23,534 (80.9)	7,136 (91.0)	1,693 (92.4)	<0.0001 (567.9)
Bungalow	5,569 (19.1)	708 (9.0)	140 (7.6)	
Habitable area (square meters)				
50	4,116 (14.1)	686 (8.7)	127 (6.9)	<0.0001 (220.2)
50	24,987 (85.9)	7,158 (91.3)	1,706 (93.1)	
Household income (Yuan/month)				
3,000	12,394 (42.6)	2,744 (35.0)	553 (30.2)	<0.0001 (232.9)
3,000	16,709 (57.4)	5,100 (65.0)	1,280 (69.8)	

*VD, vaginal delivery; CS, caesarean section with medical indications; ECS, cesarean section due to social factors*.

**Table 2:** Factors that affect ECS.

**Factor**	**Model coefficient**	**χ^2^**	***P***	**Odds ratio(95% CI)**
**Maternal factor**				
University or higher	0.893	99.8	<0.0001	2.44 (2.05–2.91)
Junior college	0.689	106.5	<0.0001	1.99 (1.75–2.27)
Certain occupations^1^	0.196	8.2	0.004	1.22 (1.06–1.39)
Age at delivery ≥35Y	0.255	7.3	0.007	1.29 (1.07–1.55)
**Family factor**				
Live in building	0.617	41.9	<0.0001	1.85 (1.54–2.23)
Single–parent	0.247	12.4	0.0004	1.28 (1.12–1.47)
Household income (≥ 3,000 Yuan/m)	0.093	10.6	0.001	1.10 (1.04–1.16)
**Fetus factor**				
Cord around neck	0.549	85.7	<0.0001	1.73 (1.54–1.95)
Incipient abortion	0.311	11.7	0.0006	1.37 (1.14–1.63)
Birth weight ≥ 4 kg	0.286	6.4	0.01	1.33 (1.07–1.66)

*VD, vaginal delivery; CS, cesarean section with medical indications; ECS, cesarean section due to social factors*.

*Certain occupations^1^ refer to teacher, doctor, public servant*.

### Rating Scale Reliability and Validity

#### Reliability

To assess response authenticity, participants were required to provide the delivery mode twice in the study questionnaire. The correlation coefficient for the correlation between the two answers was 0.993 (*P* < 0.0001, Table 3). Four rating scales were used in this study: CPRS, CBCL-PF, SNAP-IV-PF, and BRIEF-PF. The split-half reliability of these scales was 0.76 (0.70–0.80) and the Cronbach's alpha standard coefficient was 0.76, suggesting that the reliability of each scale was acceptable and the overall consistency of each scale was good.

**Table 3:** Correlation test for the two answers to the mode of delivery.

**Mode of delivery**	**Frequency (%) *n* = 38,780 (the first answer)**	**Frequency (%) *n* = 38,780(the second answer)**	**Correlation coefficient**	***P***
VD	29,103 (75.0)	29,134 (75.1)		
CS	78,44 (20.2)	7,833 (20.2)	0.993	<0.0001
ECS	1,833 (4.8)	1,813 (4.7)		
CS + ECS	9,677 (25.0)	9,646 (24.9)		

*VD, vaginal delivery; CS, cesarean section with medical indications; ECS, cesarean section due to social factors*.

#### Validity

Pearson correlation analysis showed moderate to high correlations between SNAP-IV-PF and the other three rating scales (correlation coefficients = 0.50–0.73, Table 4).

**Table 4:** Pearson correlation coefficient between different rating scales (*n* = 38,780).

	**Conners**	**CBCL**	**SNAP-IV**	**BRIEF**
Conners		0.53	0.61	0.56
CBCL	0.53		0.59	0.50
SNAP-IV	0.61	0.59		0.73
BRIEF	0.56	0.50	0.73	

### Effect of ECS on Child Psychology and Behavior

Non-parametric (Kruskal–Wallis) rank sum tests were used to evaluate the influence of ECS on child psychology and behavior. ECS affected four aspects: inattention, hyperactivity/impulsivity, social problems, and executive dysfunction. To verify the reliability of the results, stepwise multivariate logistic regression analysis was used to control for confounding factors. The results were similar to those of non-parametric (Kruskal–Wallis) rank sum testing, indicating that the results are accurate and reliable. Detailed results are listed below.

#### Attention Deficit

**Figure 3:**
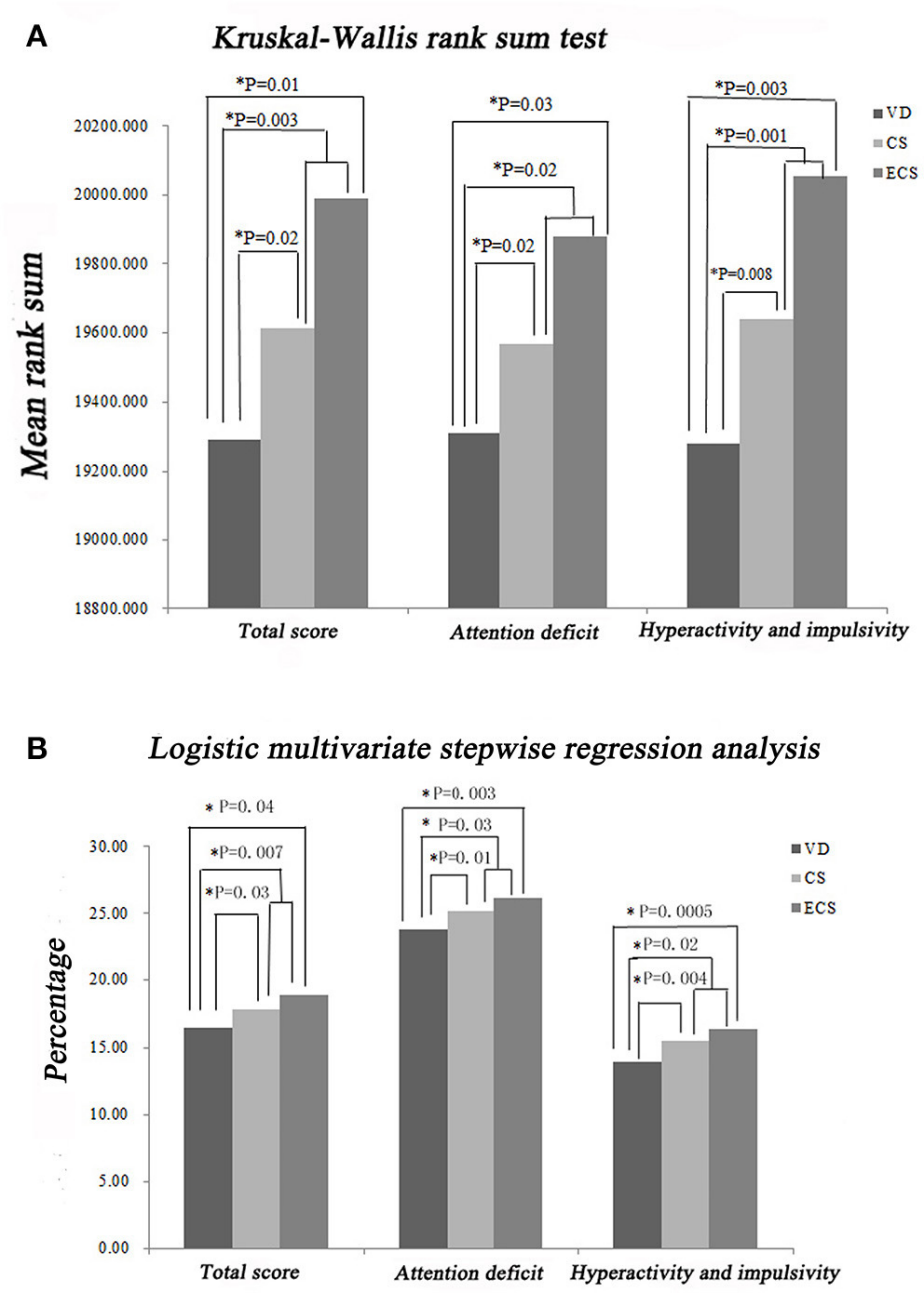
Impact of ECS on child inattention and hyperactivity/impulsivity according to the SNAP-IV-PF rating scale. **(A)** Results of Kruskal–Wallis rank sum tests. **(B)** Results of stepwise multivariate logistic regression analysis. CS, cesarean section with medical indications; ECS, cesarean section due to social factors; SNAP-IV-PF, Swanson, Nolan, and Pelham questionnaire, fourth version; VD, vaginal delivery.

The SNAP-IV-PF scores regarding inattention and hyperactivity/impulsivity varied among children with different delivery modes. Based on Kruskal–Wallis rank sum testing, the ECS group had a higher SNAP-IV-PF inattention score (*P* = 0.03, Figure 3A), compared with the VD group, indicating that children delivered by ECS are more likely to exhibit inattention. Logistic multivariate stepwise regression analysis showed that in the ECS group, the odds ratio (OR) was 1.20 in the partially adjusted analysis (adjusting for child's age, child's sex, maternal education, family relationship, and only child) compared with the VD group (*P* = 0.003, Figure 3B).

#### Hyperactivity/Impulsivity

**Figure 4:**
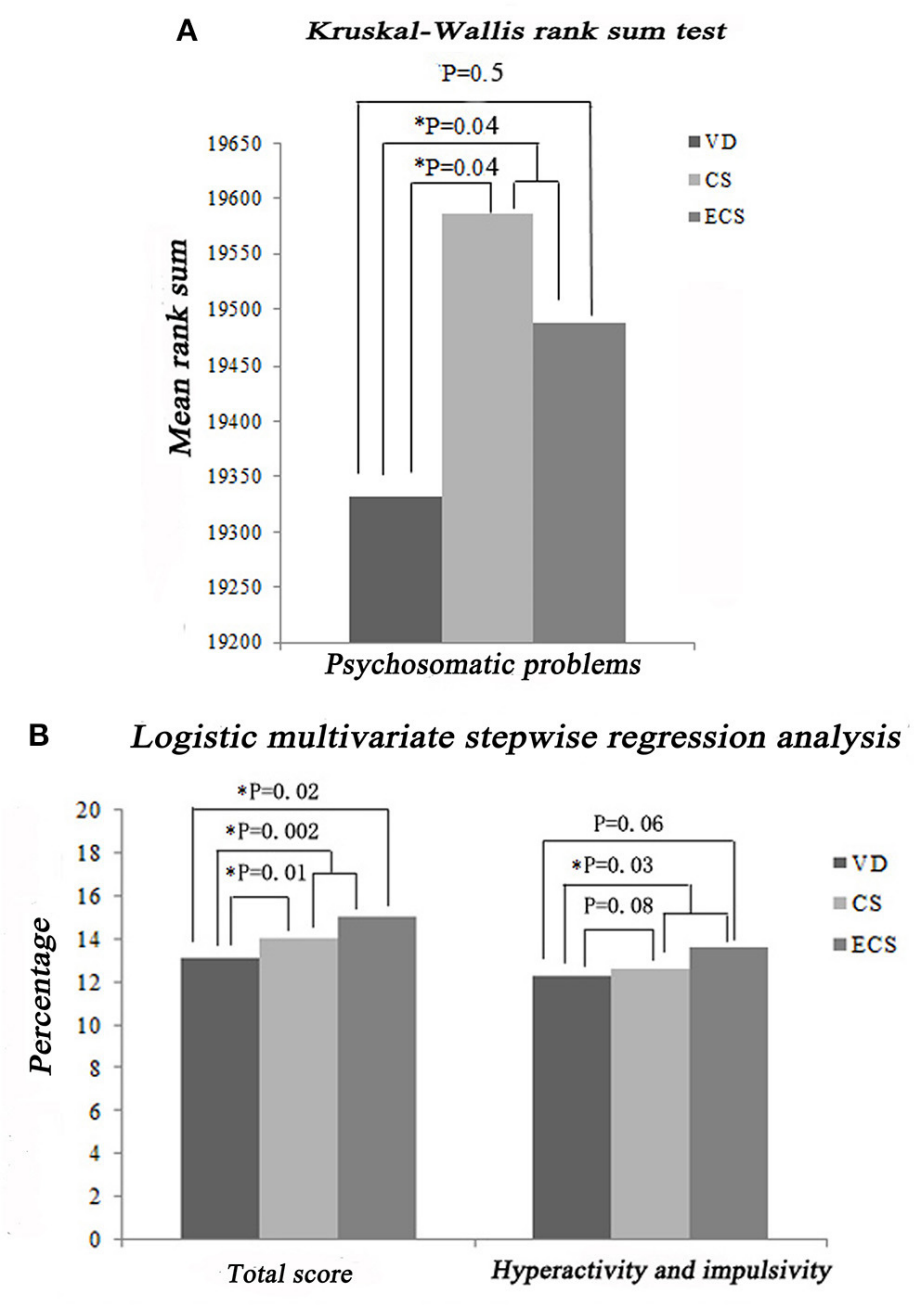
Impact of ECS on hyperactivity/impulsivity and psychosomatic problems according to the CPRS. **(A)** Results of Kruskal–Wallis rank sum tests. **(B)** Results of stepwise multivariate logistic regression analysis. CPRS, Conners' Parent Rating Scale; CS, cesarean section with medical indications; ECS, cesarean section due to social factors; VD, vaginal delivery.

SNAP-IV and CPRS were both used to assess impulsivity/hyperactivity. The Kruskal–Wallis rank sum test showed that the ECS group had a higher SNAP-IV-PF score compared with the VD group (*P* = 0.003, Figure 3A). Logistic multivariate stepwise regression analysis showed that in the ECS group, the ORs were 1.20 in the partially adjusted analyses of SNAP-IV-PF and CPRS (adjusting for child's age, child's sex, maternal education, family relationship, and only child in the SNAP-IV-PF analysis; adjusting for child's age, maternal education, family relationship, only child, single-parent family, living conditions, and habitable area in the CPRS analysis) compared with the VD group (*P* = 0.003, Figures 3B, 4B). These findings indicate that children delivered by ECS are more likely to be hyperactive/impulsive.

#### Social Problems

**Figure 5:**
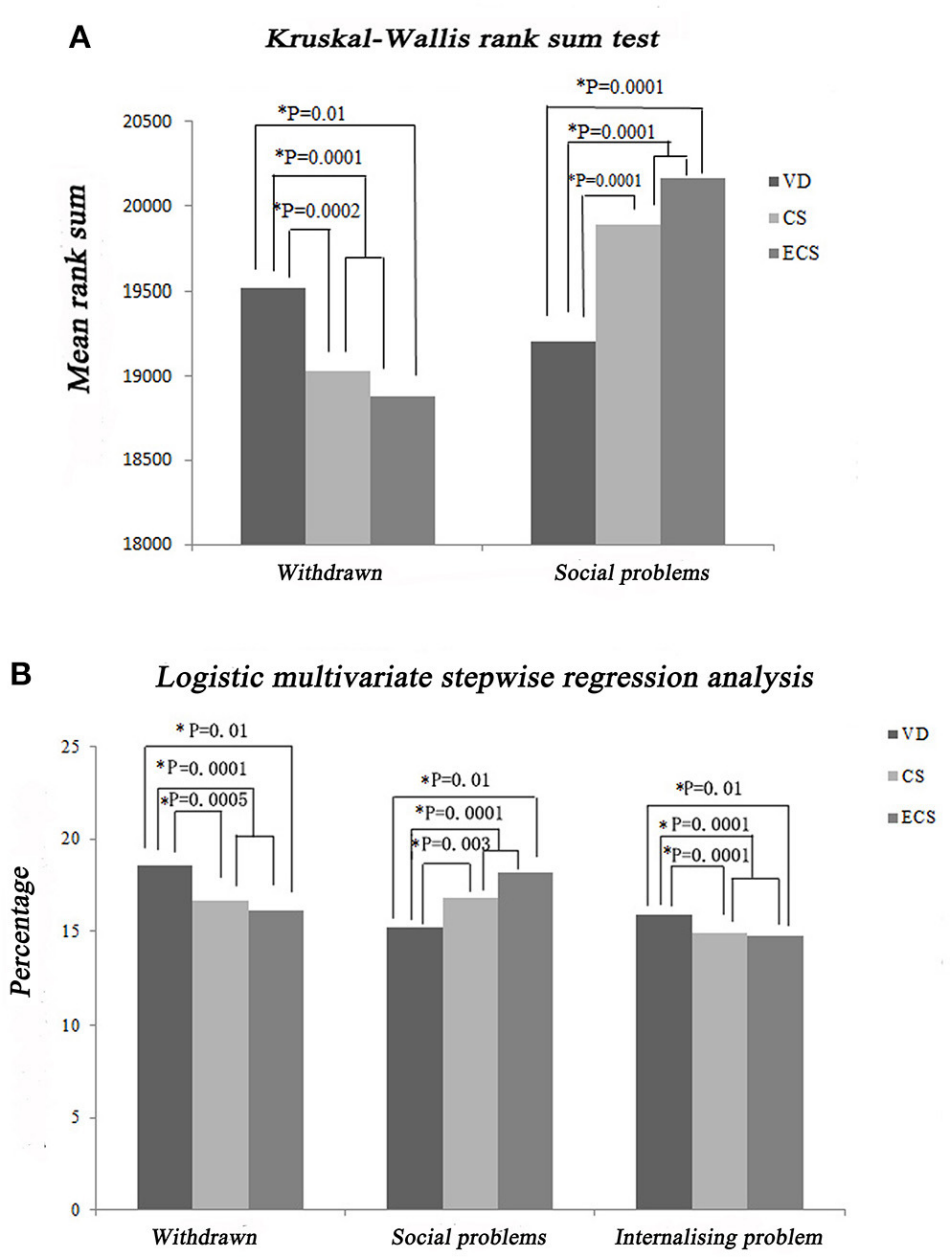
Impact of ECS on the CBCL-PF social problems, withdrawn/depressed, and internalizing problems categories. **(A)** Results of Kruskal–Wallis rank sum tests. **(B)** Results of stepwise multivariate logistic regression analysis. CBCL-PF, Child Behavior Checklist-Parent Form; CS, cesarean section with medical indications; ECS, cesarean section due to social factors; VD, vaginal delivery.

Major manifestations of social problems include acting young, clingy behavior, difficulty getting along with others, experiencing teasing, and not being liked. Kruskal–Wallis rank sum tests showed that the ECS group had a higher CBCL-PF score for the social problems category compared with the VD group (*P* = 0.0001, Figure 5A). Logistic multivariate stepwise regression analysis showed that in the ECS group, the OR was 1.30 in the partially adjusted analysis of the CBCL-PF social problems category (adjusting for child's age, child's sex, maternal education, family relationship, only child, single-parent family, living conditions, and habitable area) compared with the VD group (*P* = 0.01, Figure 5B).

#### Executive Dysfunction

**Figure 6:**
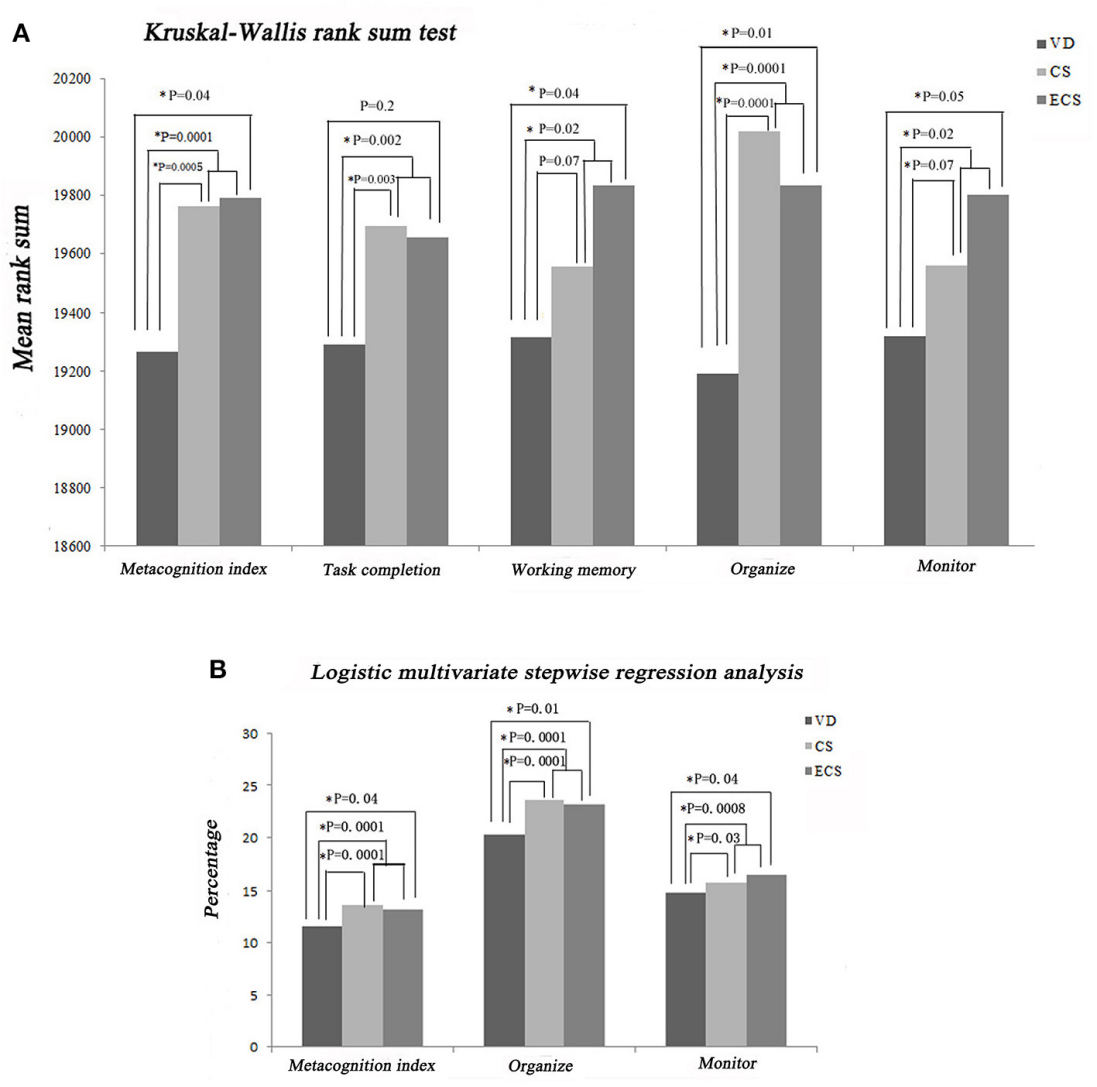
Impact of ECS on childhood executive dysfunction according to the BRIEF-PF. **(A)** Results of Kruskal–Wallis rank sum tests. **(B)** Results of stepwise multivariate logistic regression analysis. BRIEF-PF, Behavior Rating Inventory of Executive Function-Parent Form; CS, cesarean section with medical indications; ECS, cesarean section due to social factors; VD, vaginal delivery.

Executive functions were assessed using multiple scores, including the BRIEF-PF BRI subscale score (Inhibit, Shift, Emotional Control, and Monitor) and the BRIEF-PF MI subscale score (Working Memory, Plan/Organize, Organization of Materials, and Task-Monitor). Kruskal–Wallis rank sum tests showed that the ECS group had higher BRIEF-PF scores regarding MI (*P* = 0.04), Working Memory (*P* = 0.04), and Organize (*P* = 0.01) compared with the VD group (Figure 6A). Logistic multivariate stepwise regression analysis showed that in the ECS group, the ORs 1.20 in the partially adjusted analyses for MI (*P* = 0.04), Organize (*P* = 0.04), and Monitor (*P* = 0.01) (adjusting for child's age, maternal education, family relationship, only child, single-parent family, living conditions, and habitable area) compared with the VD group (Figure 6B). Higher BRIEF-PF scores indicate more severe executive function impairments.

#### Psychosomatic Problems

One of the CPRS subscales assesses psychosomatic problems including headache, stomach pain, other aches, vomiting or nausea, and bowel problems (constipation or diarrhea). The Kruskal–Wallis rank sum test showed that the CS + ECS group (involving CS with medical indications and ECS) had higher CPRS scores compared with the VD group (*P* = 0.04, Figure 4A). However, there was no significant difference between the ECS and VD groups (*P* = 0.5), indicating that medical indications for CS resulted in psychosomatic problems rather than CS itself.

## Discussion

In the present study, we found that CS due to social factors accounted for 20% CS. Many factors including higher education, some occupations (teacher, doctor, and public servant), single-parent family, household income and living condition significantly affected the choice of CS. Among them, 33 women chose CS just because of auspicious dates. We further examined the associations between delivery mode especially CS due to social factors and child psychology and behavior in a large Chinese cohort. Stepwise multivariate logistic regression analysis indicated that CS due to social factors was associated with increased risk of psychological and behavioral problems (e.g., attention deficit, hyperactivity/impulsivity, social problems, and executive dysfunction) compared with VD, and this association persisted after controlling for multiple confounding factors. Even a small increase in the risk of psychological and behavioral problems may have large impacts on society.

Previous studies have reported short-term effects of CS on newborns including delayed lung maturation (24), poor thermogenesis (25), and reduced sucking reflex and breast feeding (26). There is increasing evidence indicating that CS also has long-term impacts on child physiology. Sevelsted et al. (27) reported associations between CS and asthma, systemic connective tissue disorders, juvenile arthritis, inflammatory bowel disease, and immune deficiencies. Moreover, the Childhood Leukemia International Consortium examined the association between CS and childhood leukemia in a large sample of cases and found that pre-labor CS was associated with acute lymphoblastic leukemia (8).

In addition to long-term impacts on child physiology, CS may be related to psychology. Curran et al. (16) conducted a systematic review of the literature to assess the impact of delivery mode on ASD and ADHD and found that, compared with VD, CS was associated with modestly increased odds of ASD and possibly ADHD. However, few studies have separately described the impacts of emergency CS and ECS on ASD or ADHD. Emergency CS is performed when serious complications such as preeclampsia or fetal distress affect the mother and/or baby. These medical complications can have adverse impacts on child development. A recent study reported that the likelihood of child psychopathological problems was lowest in children born by ECS, followed by those born by spontaneous VD, whereas the highest probability was observed in those born by assisted VD (18). These findings were refuted by another study that reported that preschool children born by ECS may have increased risks of emotional disturbances and sleep problems (17). The contradictory results may be due to several study differences: some did not use population-based samples, some didn't differentiate ECS from CS with medical indications (28, 29) and did not adjust for confounders. The present study used a population-based sample and analyzed the data by non-parametric (Kruskal–Wallis) rank sum tests (for univariate analysis) and Logistic multivariate stepwise regression analysis (to control for confounding factors). Using these methods, CS due to social factors was associated with child psychological and behavioral problems.

There are several hypotheses to explain the potential relationship between CS and neurological development. First, newborns delivered by CS have different initial microbiota. Infants delivered by VD acquire bacterial communities resembling the maternal vaginal microbiota, and infants delivered by CS harbor bacterial communities similar to the skin microbiota (30). The composition of the core bacteria that we harbor during adulthood is mainly established in our first few years of life. There is a growing emphasis on the relationship between the gut microbiota and central nervous system disorders. The microbiota–gut–brain axis is composed of the brain, intestinal glands and other aspects of the gut, immune cells, and the gut microbiota. The immune system plays a key role in the microbiota–gut–brain axis (31). In an ASD mouse model created by prenatal valproic acid exposure, male offspring exhibited increased expression of neuroinflammatory markers, decreased serotonin, and disturbed social interactions compared with females (32). In another ASD mouse model created by maternal immune activation, offspring displayed both neurological and gastrointestinal symptoms associated with autism (33). Oral treatment with *Bacteroides fragilis* corrects gut permeability and resolved behavioral symptoms of ASD in offspring, suggesting that there are possible associations among the microbiota, immune system, and ASD (34). In addition, the stress of VD primes the hypothalamic–pituitary–adrenal axis and immune system, which enables offspring to deal with future insults (35). Furthermore, after ECS, the infant is separated from the mother after birth, which may have a negative impact on early bonding and attachment. Another possible explanation is that delivery by CS due to social factors is associated with changes in dopaminergic function and biochemistry (36), which alters how stress affects dopaminergic function.

There are several limitations to this study that need to be considered. Firstly, study didn't account for such potentially confounding variable as gestational age of the fetus, while gestational age in the case of ECS tends to be lower as obstetricians try to avoid spontaneous labor. Secondly, data analysis didn't differentiate the data related to the different types of VD such as assisted VD. However, despite this potentially obscuring factor associated with potential harm to the baby, statistical significance with regards to outcomes still remained. This study has several strengths. First, it is one of the largest observational studies to address the issue of effect of ECS on child psychology and behavior. Second, participants in the study were required to provide a delivery mode twice thus strengthening data on this key variable. Third, multiple rating scales and statistical methods are applied to measure the variables in this study.

Collectively, the evidence suggests that CS due to social factors is associated with increased odds of a wide spectrum of psychological and behavioral problems which may have large impacts on society. These findings improve our understanding of CS, help mothers make informed choices regarding the delivery mode and contribute to decreasing CS rate in China. We must keep in mind that everything has its own regular pattern, we should comply with it as much as possible. Further research is needed to confirm these findings in other cohorts and identify potential mechanisms.

## Data Availability Statement

The original contributions presented in the study are included in the article/Supplementary Material, further inquiries can be directed to the corresponding author/s.

## Ethics Statement

Ethical review and approval was not required for the study on human participants in accordance with the local legislation and institutional requirements. Written informed consent to participate in this study was provided by the participants' legal guardian/next of kin.

## Author Contributions

L-PZ conceived the study. X-YS, JW, W-NZ, JJ, X-YL, QL, and BW collected, interpreted the data and wrote the original draft of the manuscript. L-PZ reviewed and edited the manuscript. MZ performed the statistical analyses. All authors read and approved the final manuscript.

## Conflict of Interest

The authors declare that the research was conducted in the absence of any commercial or financial relationships that could be construed as a potential conflict of interest.
